# Statins for Renal Patients: A Fiddler on the Roof?

**DOI:** 10.1155/2012/806872

**Published:** 2012-10-22

**Authors:** Anabela Malho Guedes, Pedro Leão Neves

**Affiliations:** Serviço de Nefrologia, Hospital de Faro, 8000 Faro, Portugal

## Abstract

Atherosclerotic cardiovascular disease is the main cause of morbidity and mortality in chronic kidney disease patients. There is a raft of evidence showing that in the general population dyslipidaemia is associated with an increased risk of cardiovascular events, as well as with a greater prevalence of chronic kidney disease. Consequently, the use of statins in the general population with dyslipidaemia is not controversial. Nevertheless, the benefits of statins in patients with chronic kidney disease are more elusive. The authors review the possible effects of statins on the progression of renal disease and cardiovascular events in chronic kidney disease patients.

## 1. Introduction

Atherosclerotic cardiovascular disease is the main cause of morbidity and mortality in chronic kidney disease patients [1, 2], with many factors such as high blood pressure, dyslipidaemia, increased oxidative stress and inflammation, diabetes, and mineral metabolism imbalances contributing to this [3]. As the pathophysiology of atherosclerosis and glomerulosclerosis is similar [3], it is not surprising to find they have the same risk factors [3]. There is a raft of evidence showing that in the general population dyslipidaemia is associated with increased risk of cardiovascular events [4], as well as with a greater prevalence of chronic kidney disease [5, 6]. Consequently, the use of statins in the general population with dyslipidaemia is not controversial. Nevertheless, the benefits of statins in patients with chronic kidney disease are more elusive. The aim of this work is to review the possible effects of statins on the progression of renal disease and cardiovascular events in chronic kidney disease patients.

## 2. Dyslipidaemia and Chronic Kidney Disease

### 2.1. Effect on Renal Function

It is still uncertain whether dyslipidaemia itself causes kidney disease progression or whether kidney impairment and proteinuria are responsible for both renal disease progression and dyslipidaemia [7]. Evidence suggests that in the early stages of renal impairment, dyslipidaemia increases the likelihood of CKD and may also accelerate its progression by promoting intrarenal atherogenesis and cellular impairment in the microvasculature [8]. The role of dyslipidaemia in promoting kidney damage has been shown in experimental models. Rats fed a diet rich in cholesterol and fat exhibited increased numbers of glomeruli with sclerotic foci and of glomeruli with lipid deposits. 

The severity of the hypercholesterolemia correlates with proteinuria. In rats with kidney disease caused by unilateral nephrectomy, such diet augmented the glomerular lesions in the remaining kidney [9]. Renal biopsies from patients with glomerular disease indicate that lipoproteins accumulate in both glomerular and mesangial cells and within the mesangial matrix, and oxidised lipids are frequently found in biopsy specimens from patients with renal disease [8, 10]. The presence of lipids in renal cells exerts a nephrotoxic effect and accelerates glomerulosclerosis, by upregulating intracellular signalling pathways involved in inflammatory and fibrogenic responses, both of which are implicated in progressive renal injury [11, 12].

Samuelsson et al., in a small study with 73 adult nondiabetic patients with primary chronic renal disease, showed that lipoprotein abnormalities can contribute to the progression of kidney failure in CKD patients and demonstrated an association between progression of renal disease and hyperlipidaemia [11]. The Physicians' Health Study followed 4483 healthy men, at the outset, for a mean of 14.2 years. After adjustment for potential confounding factors (cardiovascular risk factors and development of hypertension and cardiovascular disease), men in the highest quartile of total cholesterol/high-density lipoprotein cholesterol ratio had a 92% higher risk of developing CKD than those in the lowest quartile [13]. 

The Helsinki Heart Study documented an association between dyslipidaemia and progressive kidney disease in 2702 middle-aged dyslipidaemic men. Renal function deteriorated by a mean of 3% over 5 years, and although hypertension accelerated this change, the decline was faster by 20% in men with an LDL : HDL ratio >4.4 than in men with a ratio <3.2. After multiple regression analyses, the only measures having a significant effect on the observed phenomenon were an increased LDL : HDL ratio (negative outcome) and an increased HDL-C level (protective outcome) [5, 8].

### 2.2. Effect on Cardiovascular Disease

The lipid profile is influenced by the severity of renal dysfunction and the presence of proteinuria. Patients with CKD have, in general, lower levels of HDL, LDL, and total cholesterol and higher levels of triglycerides. There is a clustering of low HDL and elevated Lp(a) and TG-rich ApoB containing VLDL and IDL [14]. Patients with nephrotic syndrome have increased levels of all Apo-B containing lipoproteins (VLDL, IDL, and LDL) and decreased levels of HDL [14]. Although dyslipidaemia has an important role in CKD-associated atherosclerosis, the relationship between cardiovascular (CV) events and cholesterol levels in this particular population is not straightforward. Almost 150 years ago, Virchow described atherosclerosis as an inflammatory disease.

In the last decades the medical literature confirmed that oxidative stress and inflammation are major players in the pathogenesis of atherosclerosis [15], and more recently, it has been well described that chronic kidney disease is characterised by increased oxidative stress and inflammation [16, 17]. Despite normal or low cholesterol levels, in renal patients there is a greater proportion of the more atherogenic LDL oxidised form [14]. This fact can in part explain why in CKD patients there is a strong relationship between inflammatory markers and CV hard endpoints and patients with low cholesterol levels may have poor outcomes. This inverse relationship between cholesterol levels and cardiovascular events, in marked contrast to the general population, is called reverse epidemiology. Renal patients with low cholesterol levels tend to be malnourished and more “inflamed.” This association of malnutrition, inflammation and atherosclerosis was well described by Stenvinkel et al. and called the MIA syndrome [18].

## 3. Does the Use of Statins Lower the Progression of Renal Disease?

If dyslipidaemia promotes renal injury, then reducing dyslipidaemia should slow or prevent the progression of CKD. Experimental models show that statins decrease the severity of glomerular damage and preserve renal function. For example, New Zealand rabbits fed a diet rich in cholesterol became hypercholesterolaemic, with evidence of endothelial dysfunction in renal segmental arteries as well as glomerular hypertrophy and diffuse glomerulosclerosis. In this model, atorvastatin attenuated the increase in plasma cholesterol and prevented renal artery endothelial dysfunction, glomerular hypertrophy, and most of the glomerulosclerosis [19]. Data on the efficacy of statin therapy in patients with renal dysfunction is limited, as these patients were excluded from early statin trials [9]. The majority of these data come from post hoc analyses or from patients randomised for cardiovascular primary endpoint trials [20] (Table 1).

### 3.1. Statins and Proteinuria

The presence of proteinuria is an indicator of kidney disease with an increased probability of progressive kidney failure and is associated with faster loss of GFR compared with little or no proteinuria [20]. In a meta-analysis by Sandhu et al., the effects of statins on proteinuria and albuminuria were evaluated in 9 studies (350 participants) and 7 studies (904 participants), respectively. When proteinuria and albuminuria were considered separately, statin therapy did not significantly influence the rate of change in urinary protein or albumin excretion. However, when considered together, statins significantly reduced urinary protein and albumin excretion compared with controls (standardised mean difference between treatments, −0.58 units of SD) [21]. 

A meta-analysis by Douglas et al. evaluated 15 studies involving a total of 1,384 patients and examined the proportional reduction in proteinuria with the use of statins. It was shown that statins reduced albuminuria and proteinuria in 13 of the 15 studies. The reduction of excretion was greater among studies with greater baseline albuminuria or proteinuria. More specifically, 440 patients with albuminuria ≥30 mg/day showed a 48% reduction of albuminuria relative to placebo [22]. 

Another meta-analysis of six randomised placebo control trials including 311 patients showed that compared to placebo, statins reduced proteinuria significantly (−0.73 g/24 h) [23]. A differential effect on proteinuria has also been suggested, with different statins. PLANET I (Prospective Evaluation of Proteinuria and Renal Function in Diabetic Patients with Progressive Renal Disease) and PLANET II (Evaluation of Proteinuria and Renal Function in Nondiabetic Patients with Progressive Renal Disease), two related randomised, double-blind, parallel-group, multinational, multicentre, phase IIb trials, evaluated the effects of atorvastatin and rosuvastatin on urinary protein excretion and kidney function from baseline to week 52 in hypercholesterolaemic diabetic and nondiabetic patients, respectively. 

In PLANET I, atorvastatin (80 mg) significantly reduced proteinuria by about 15%, whereas rosuvastatin (10 or 40 mg) had no significant effect on proteinuria. In PLANET II, atorvastatin (80 mg) reduced proteinuria by 23.8% (*P* = 0.0056) [20]. Discrepant findings have been reported in some studies, as short-term therapy with statins, particularly at high doses, can induce proteinuria. Urinary protein electrophoresis indicated that most of the protein excreted had a lower molecular weight than albumin, suggesting that this may be due to reduced receptor-mediated endocytosis in proximal tubular cells [9, 20, 24]. This may help explain why statins can transiently increase protein excretion and simultaneously protect against renal damage. 

The National Lipid Association Statin Safety Assessment Task Force reported that “proteinuria is at least possible with all statins at some concentration, but is more likely to be seen with statins that are potent inhibitors of HMG-CoA reductase.” The report concludes that statin-induced proteinuria is not associated with either renal impairment or renal failure [9, 25]. Accordingly, proteinuria is not a reliable surrogate endpoint for important renal outcomes, when we are assessing the effects of statins in CKD patients. 

### 3.2. Statins and CKD Progression

Two trials—the Greek Atorvastatin and Coronary Heart Disease Evaluation (GREACE) study and the Aggressive Lipid-Lowering Initiation Abates New Cardiac Events (ALLIANCE) study—evaluated atorvastatin therapy versus usual care in patients with coronary artery disease (CAD). In both studies, creatinine clearance declined by an average of 4.4% during 4 years in patients assigned to usual care. In contrast, patients assigned to atorvastatin therapy had an 11.6% increase in creatinine clearance in the GREACE study. In the atorvastatin group in the ALLIANCE study, although creatinine clearance did not improve, no decline was seen. The difference between treatments was highly significant in both studies (*P* < 0.0001) [26, 27].

A *Post Hoc* analysis of the Cholesterol and Recurrent Events (CARE) trial, a secondary prevention trial of pravastatin versus placebo, showed a significant difference in the rate of decline with pravastatin (2.5 mL/min/1.73 m^2^ per year slower than in placebo recipients; *P* < 0.0001) in those with severe CKD at baseline (estimated GFR <40 mL/min/1.73 m^2^) [28]. The aforementioned meta-analysis by Sandhu et al. included 27 randomised trials (39,704 participants) and concluded that, compared with no treatment, statins slowed the loss of GFR by a mean of 1.22 mL/min/year [21]. 

The Study of Heart and Renal Protection (SHARP), a prospective, randomised, controlled study, assessed outcomes in approximately 6300 CKD patients given combination simvastatin/ezetimibe therapy. Concerning the secondary endpoint of progression to ESRD in SHARP, no difference was seen between groups. In fact, one third of the patients in both arms progressed to dialysis or transplantation [29]. Concerning the evaluation of renal function, two prospective, controlled, randomised studies—the aforementioned PLANET I and PLANET II studies—involved 325 patients with type 1 or 2 diabetes with a mean eGFR of 71.2 mL/min/1.73 m^2^, and 237 patients with a mean eGFR of 74.9 mL/min/1.73 m^2^ at baseline, respectively. Recent results showed that patients on atorvastatin lost about 1 to 2 mL/min per 1.73 m^2^ over 52 weeks, those on rosuvastatin 10 mg/day lost about 4 mL/min per 1.73 m^2^ and those on rosuvastatin 40 mg/day lost close to 8 mL/min per 1.73 m^2^ [30–32]. 

In nondiabetic patients (PLANET II), the effects of the treatments on kidney function were slightly less pronounced. There was a significant decline in eGFR with rosuvastatin 40 mg/day but not in the other two treatment groups [30–32]. Atorvastatin and rosuvastatin exerted different effects on proteinuria and renal function. The differential effects on proteinuria and eGFR in the treatment groups were not a result of differences in lipid lowering. All the treatments lowered total and LDL cholesterol, and there were no significant differences in the amount of lipid lowering [32]. One big question remaining is whether atorvastatin is actually protecting the kidneys or whether rosuvastatin is damaging them [32]. 

The available data and the evidence are inconclusive to answer the question if statins slow the kidney disease progression. It is premature to recommend statin therapy for renal protection alone. Additional prospective, randomised trials are also needed to determine whether statins are truly renoprotective. Addressing safety, the use of high doses of statins in CKD patients has largely been demonstrated to be safe and well tolerated [20, 23].

## 4. Does the Use of Statins Lower Cardiovascular Disease Burden on CKD Patients?

Statins improve the lipid profile in renal patients and exert several pleiotropic effects without major adverse consequences. However, respecting cardiovascular outcomes is critical to the timing of the initiation of the therapy. In the SHARP trial the use of simvastatin plus ezetimibe versus placebo reduced major atherosclerotic events (coronary death, myocardial infarction, nonhaemorrhagic stroke, or any revascularisation) only in the group of patients not on dialysis (risk reduction of 20.2%). In dialysis patients the difference did not reach statistical significance [29]. 

This observation corroborates the result of the Prospective Pravastatin Pooling Project that included three large trials: CARE, WOSCOPS, and LIPID studies [33]. Pravastatin also reduced significantly the incidence of myocardial infarction, coronary death, and coronary revascularisation by 23%, only in patients with moderate renal insufficiency [33]. Recently, we also could find, in an observational study, that statins plus vitamin D reduced cardiovascular mortality in predialysis patients (stages 4 and 5) [34]. 

### 4.1. CKD Patients on Hemodialysis

On the other hand, it is known from several randomised controlled trials (SHARP, 4D, and AURORA) [29, 35, 36] that the use of statins in patients already under renal replacement therapy was not protective in terms of mortality and cardiovascular events.

The apparent lack of benefit of using statins in patients in dialysis can have quite a few explanations: the existence of other pathogenic pathways contributing to cardiovascular disease, the high mortality of dialysis patients due to sudden death and cardiomyopathy, situations not preventable by statins, or just because it is too late to interfere with the natural history of atherosclerosis. Moreover, inflammation can play an important role in the dialysis population. In the JUPITER study, the administration of rosuvastatin in patients with mild dyslipidaemia but with high CRP levels reduced the number of cardiovascular events [37]. 

This finding was not unexpected, since statins have an anti-inflammatory effect. This fact can also explain the negative results in the 4D and AURORA trials, because in these studies the CRP levels remained high despite the use of atorvastatin or rosuvastatin, respectively [38].

Nevertheless, in a recent post hoc analysis of the 4D trial, it was shown that atorvastatin decreased the risk of fatal and nonfatal cardiac events in dialysis diabetic patients with high levels of LDL cholesterol levels [39].

## 5. Conclusion

Statins may have antiproteinuric effects, although the clinical significance of these benefits remains uncertain. The available data and the evidence are inconclusive to answer the question if these agents slow the kidney disease progression. It is premature to recommend statin therapy for renal protection alone.

Regarding prevention of cardiovascular disease, there is a strong clinical evidence of benefit in using statins in early stages of CKD, but there is no clear advantage to its use in dialysis patients, a particular population with a huge cardiovascular risk. Nevertheless these drugs have several pleiotropic effects, and remembering the musical “The Fiddler on the Roof” and the song “If I Were a Rich Man…” maybe we can also use statins in selected dialysis patients; namely, in those with higher LDL cholesterol levels, their use may be advantageous. 

## Figures and Tables

**Table 1:** Overview of major statin studies.

Study		Patient population	Followup	Treatment	Outcome	Results
Overview of renal outcomes						

GREACE	Post hoc subgroup analysis	1,600 patients with dyslipidemia and CAD	3 years	Atorvastatin 10–80 mg/day or usual medical care	Rate of kidney function decline	CrCl had a 12% increase in atorvastatin group (*P* < 0.0001) CrCl had a 5.2% decrease in patients not treated with statins (*P* < 0.0001) CrCl had a 4.9% increase in the usual care group on various statins

ALLIANCE	Post hoc subgroup analysis	2,442 patients with dyslipidemia	4 years	Atorvastatin 10–80 mg/day or usual medical care	Rate of kidney function decline	CrCl did not change in the atorvastatin group versus baseline CrCl declined by 4.4% in the usual care group (*P* = 0.0001 versus baseline)

CARE	Post hoc subgroup analysis	3,384 individuals of whom 690 (20.4%) had GFR < 60 mL/min per 1.73 m^2^	4 years	Pravastatin 40 mg/day versus placebo	Change in GFR	The decline in the pravastatin group versus placebo was nonsignificant In patients with GFR < 40 mL/min per 1.73 m^2^, the rate of change in the pravastatin versus placebo group was 2.5 mL/min per 1.73 m^2^/year slower (95% CI: 1.4–3.6; *P* = 0.0001)

SHARP	Randomized double blind, multicenter trial	9,270 participants, including 3000 receiving hemodialysis	4.9 years	Ezetimibe 10 mg/day + simvastatin 20 mg/day versus placebo versus simvastatin 20 mg/day	ESRD, major atherosclerotic events	17% reduction in major atherosclerotic events No difference of progression to ESRD

PLANET I	Randomized double blind, multicenter trial	325 patients with diabetes who had proteinuria and hypercholesterolemia	1 year	Rosuvastatin 10 mg/day or rosuvastatin 40 mg/day versus atorvastatin 80 mg/day	Change in urinary protein excretion (urinary protein/ creatinine ratio)	Atorvastatin significantly reduced proteinuria by about 15% rosuvastatin had no significant effect on proteinuria Patients on atorvastatin lost 1 to 2 mL/min per 1.73 m^2^, those on rosuvastatin 10 mg/day lost 4 mL/min per 1.73 m^2^, and those on rosuvastatin 40 mg/day lost 8 mL/min per 1.73 m^2^ over 52 weeks

PLANET II	Randomized double-blind, multicenter trial	220 patients without diabetes who had proteinuria and hypercholesterolemia	1 year	Rosuvastatin 10 mg/day or rosuvastatin 40 mg/day versus atorvastatin 80 mg/day	Change in urinary protein excretion (urinary protein/ creatinine ratio)	Atorvastatin reduced proteinuria by 23.8% (*P* = 0.0056) Significant decline in GFR with rosuvastatin No significant difference in the amount of lipid lowering was reported among the treatment groups

Strippoli et al. [23]	Meta-analysis	11 studies, 548 patients		Different statins	Change in GFR	Statins did not improve GFR

Sandhu et al. [21]	Metaanalysis	27 studies (21 with data for GFR), 39,704 participants		Different statins	Change in GFR	Statins slowed the loss of GFR by a mean of 1.22 mL/min/year; 95% CI: 0.44–2.00 In studies of CVD, patients were slower than controls (0.93 mL/min/year, 95% CI: 0.10–1.76), with statistical significance

Douglas et al. [22]	Metaanalysis	15 studies, 1,384 patients		Different statins	Change in urinary protein excretion	Statins reduced albuminuria and proteinuria in 13 studies The reduction of excretion was greater among studies with greater baseline albuminuria or proteinuria

Overview of cardiovascular outcomes in patients with kidney disease						

Pravastatin Pooling Project (WOSCOPS, CARE and LIPID)	Randomized double-blind, multicenter trial	4,491 patients with or without CAD and with moderate CKD (GFR, 30–60 mL/min/1.73 m^2^)		Pravastatin 40 mg/day versus placebo	Time to MI, coronary death, or PCR	Significant reduction in primary outcome in statin-treated patients, with moderate CKD (HR: 0.77, 95% CI: 0.68–0.86); reduction in total mortality in treated patients

4D Study	Randomized double blind, multicenter trial	1,255 hemodialysis patients, with type 2 diabetes	4 years	Atorvastatin 20 mg/day versus placebo	Composite of cardiac death, nonfatal MI, and stroke	No significant difference in primary endpoint with statin treatment, but increased risk for fatal stroke (*P* = 0.04)

AURORA	Randomized double blind, multicenter trial	2,776 patients receiving long-term hemodialysis	3.2 years	Rosuvastatin 10 mg/day versus placebo	Composite of cardiac death, nonfatal MI, and stroke	Rosuvastatin lowered the LDL level (*P* < 0.0001) but had no significant effect on primary endpoint

GREACE: Greek Atorvastatin and Coronary Heart Disease Evaluation; ALLIANCE: Aggressive Lipid-Lowering Initiation Abates New Cardiac Events; CARE: Cholesterol And Recurrent Events; SHARP: Study of Heart and Renal Protection; PLANET: Prospective Evaluation of Proteinuria and Renal Function in Diabetic Patients; WOSCOPS: West of Scotland Coronary Prevention Study; LIPID: Long-Term Intervention with Pravastatin in Ischaemic Disease; 4D Study: Die Deutsche Diabetes Dialyse Studie; AURORA: A Study to Evaluate the Use of Rosuvastatin in Subjects on Regular Hemodialysis; CAD: coronary artery disease; CrCl: creatinine clearance; GFR: glomerular filtration rate; ESRD: end-stage renal disease; CVD: cardiovascular disease; CKD: chronic kidney disease; PCR: percutaneous coronary revascularization.
